# Brain aging among individuals with trigeminal neuralgia

**DOI:** 10.1016/j.ynpai.2025.100201

**Published:** 2025-10-17

**Authors:** Yenisel Cruz-Almeida, Pedro A. Valdes-Hernandez, Yun Liang, Mingzhou Ding, John K. Neubert

**Affiliations:** a Pain Research & Intervention Center of Excellence, University of Florida, Gainesville, FL, United States; b Department of Community Dentistry and Behavioral Science, College of Dentistry, University of Florida, Gainesville, FL, United States; c Department of Neuroscience, College of Medicine, University of Florida, Gainesville, FL, United States; d Department of Health Outcomes & Bioinformatics, College of Medicine, University of Florida, Gainesville, FL, United States; e Department of Biomedical Engineering, University of Florida, Gainesville, FL, United States; f Department of Biomedical Sciences, College of Dentistry, Texas A&M University, Dallas, TX, United States

**Keywords:** Trigeminal neuralgia, Pain, Aging, Brain, Brain age, Brain age gap

## Abstract

Trigeminal neuralgia (TN) is a complex orofacial neuropathic pain condition with limited understanding of underlying mechanisms and therapeutic options. Emerging evidence suggests the involvement of the brain in persons with TN including widespread brain changes when employing a widely used brain aging biomarker that estimates a predicted brain age difference or brain age gap. The aim of the present cross-sectional study was to assess the predicted brain age difference (brain-PAD) or brain age gap across two TN subtypes (classical TN, and secondary/idiopathic TN) in comparison with age-and sex-matched pain-free controls and its association with several clinical and psychological characteristics. Thirty-four individuals diagnosed with Classical TN, 17 diagnosed with secondary/idiopathic TN were age- and sex-matched to pain-free controls (n = 54). All participants underwent a T1 brain MRI and completed clinical and psychological measures. There were significant differences in brain-PAD among TN subtypes (ANCOVA p = 0.0078, effect size f^2^ = 0.28^2^), with individuals diagnosed with Classical TN having a brain-PAD significantly greater than the controls by 3.87 years (p = 0.01, Bonferroni-corrected). There were no significant brain-PAD differences between secondary/idiopathic TN and pain-free controls. Brain-PAD had a significant positive association with both pain catastrophizing (p < 0.05) and pain-related anxiety (p < 0.05), but no significant association with disease duration (p < 0.05) or usual pain intensity (p < 0.05) in persons with classical TN. The results were similar using a second brain aging biomarker. We report here accelerated brain aging processes in individuals with classical TN, but not in persons diagnosed with secondary/idiopathic TN. Our study replicates previous findings and adds to the literature that accelerated brain aging may not occur across all TN subtypes. Given the increased use of MRI for TN diagnostics, combined with our own recent work deriving brain aging biomarkers from clinical-grade scans, future studies within clinical settings are feasible and may help understand this debilitating condition.

## Highlights

• Individuals diagnosed with Classical TN showed an older brain age compared to pain-free controls.
• There were no significant brain aging differences between secondary/idiopathic TN and pain-free controls.
• Brain aging was significantly associated with both pain catastrophizing and pain-related anxiety, but not with disease duration or usual pain intensity.
• Our study replicates previous findings and adds to the literature that accelerated brain aging may not occur across all TN subtypes.

## Introduction

Trigeminal neuralgia (TN), previously known as tic douloureux, is a complex neuropathic pain condition characterized by recurrent brief episodes of electric, lancinating, shock-like pains affecting the structures innervated by the fifth cranial nerve. The prevalence of TN increases with age and women are disproportionately affected. The International Headache Society (IHS) and International Association for the Study of Pain (IASP) established a new classification system for TN, which includes subtypes of classical TN, secondary TN, and/or idiopathic TN (Olesen 2018). This classification scheme aims to incorporate the current understanding of the pathophysiology of the disease with clinical presentation to better aid clinicians in effectively diagnosing TN subtypes and thereby guiding treatments based on the diagnosis. Classical TN is generally driven by compression of the nerve root and typically responds to surgical intervention (e.g., microvascular decompression (MVD)), while secondary/idiopathic TN has no clear cause in most cases and may be more refractory to surgery. Given the lack of therapeutic options, research is urgently needed to understand the underlying neurobiology of the various TN subtypes to identify novel targets and improve treatments for this complex, debilitating condition.

Emerging evidence supports changes in brain structure and function of specific cortical and subcortical regions in TN patients compared to controls (Wang et al., 2017; Tang et al., 2020; Zhang et al. 2020; Liu et al., 2022; Xu et al., 2022; Xiong et al., 2023). Further, individuals with TN also appear to have widespread brain changes when employing a widely used brain aging biomarker (Hung et al. 2022). However, the latter study did not differentiate between TN subtypes, nor examined the association between the brain age biomarker with measures of pain and psychosocial function. It is not currently clear whether accelerated brain aging processes occur across all TN diagnoses or only a specific subtype and the relationship with various clinical characteristics. Given the need to find potential sensitive biomarkers that can capture the cumulative neurobiological impact of chronic pain on brain structure, tools for characterizing the differential burden of TN subtypes on brain health, and potential prognostic indicators that may help identify individuals at risk for more severe disease trajectories or cognitive complications, the aim of the present study was to compare predicted brain age differences or brain age gap across two discrete TN subtypes (classical TN, and secondary/idiopathic TN) and age-and sex-matched pain-free controls. Given that classical TN is characterized by trigeminal nerve compression with alterations in nerve myelination and atrophy (Hilton et al., 1994; Rappaport et al., 1997; Devor et al., 2002; Love et al., 2001; Marinković et al., 2009), we hypothesized that this subtype would exhibit accelerated brain aging compared to other TN subtypes and healthy pain-free controls. We also hypothesized that worse clinical and psychological characteristics would be associated with an older brain.

## Materials and methods

### Participants

The present investigation was performed at the University of Florida (UF) Health Sciences Center (UFHealth) and the Evelyn F. and William L. McKnight Brain Institute (MBI) at the University of Florida. The study was carried out in accordance with the Declaration of Helsinki and the study conforms to the STROBE Guidelines. Screening was done either in person or via the telephone for all participants. All participants provided verbal and written informed consent during their visit to UFHealth. A trained study coordinator read a standard script explaining all the study procedures to all subjects.

#### Individuals with trigeminal neuralgia

Participants with trigeminal neuralgia (TN) were recruited from referrals provided by the Facial Pain Research Foundation as part of a neuroimaging study investigating the functional brain mechanisms of trigeminal neuralgia (Liang et al., 2023; Liang et al., 2024). The study was approved by the WCG Institutional Review Board (IRB). Participants with TN completed the Oregon Health Science University (OHSU) Trigeminal Neuralgia –Diagnostic Questionnaire. A board-certified Orofacial Pain Specialist (JKN) completed a focused medical history, a trigeminal cranial nerve exam, and a physical examination.

Inclusion criteria

• Men and women.
• Over 18 years of age.
• American Society of Anesthesiologists (ASA) status 1, 2, or 3, deemed in good general health.
• Self-reported average usual pain intensity at screening using a Visual Analogue Scale of 30 out of 100.
• Trigeminal neuralgia diagnosis by a physician using the International Headache Society (IHS) Disorders criteria (Arnold, 2018) that was verified with physicians by the study staff:
Recurrent paroxysms of unilateral facial pain in the distribution(s) of one or more divisions of the trigeminal nerve, with no radiation beyond, and fulfilling criteria B and C.
with the following pain characteristics: lasting from a fraction of a second to two minutes, of severe intensity, electric shock-like, shooting, stabbing or sharp in quality, precipitated by innocuous stimuli within the affected trigeminal distribution, and not better accounted for by another ICHD-3 diagnosis.
• Participants included having symptoms as purely paroxysmal pain or having intermittent paroxysmal bouts with a more continuous background of burning and/or aching pain.

Exclusion criteria

• Participants diagnosed with trigeminal neuralgia attributed to space-occupying lesion (ICHD-3 code: 13.1.1.2.2) or other cause (e.g., multiple sclerosis, ICHD-3 code: 13.1.1.2.3), painful trigeminal neuropathy (ICHD-3 code: 13.1.2), trigeminal post-herpetic neuralgia (ICHD-3 code: 13.1.2.2), trigeminal neuropathic pain (ICHD-3 code: 13.1.2.4), and idiopathic painful trigeminal neuropathy (ICHD-3 code: 13.1.2.5).
• ASA status 4–5 and Emergency operation.
• Presence of chronic disease (e.g., cardiovascular disease, liver disease, kidney disease, diabetes, etc.), other than trigeminal neuralgia.
• Pregnant females.

Eligible subjects completed a study packet followed by an MRI scan. The study packet contained the following self-reported measures consistent with the biopsychosocial experience of pain:

a. **Usual Pain Intensity:** Participants were asked to rate their daily pain experienced during the past month. Subjects indicated on a 100 mm visual analog scale (VAS) anchored on the left with “no pain sensation” and on the right with “most intense pain sensation imaginable”.
b. **Pain Anxiety Symptom Scale (PASS):** Participants were asked to rate the frequency of responses to pain on a 0 (never) to 5 (always) rating scale. The PASS contains 4 subscales characterizing different responses to pain: cognitive, escape/avoidance, fear, and physiological anxiety (McCracken et al. 1992).
c. **Beck Depression Inventory-II (BDI-II):** The BDI-II measures the severity of depressive symptoms. The inventory is composed of 21 items relating to symptoms such as hopelessness and irritability, cognitions such as guilt or feelings of being punished, as well as physical symptoms such as fatigue, weight loss, and lack of interest in sex (Dozois et al. 1998).
d. **Pain Catastrophizing Scale (PCS):** The PCS consists of 13 statements containing a number of thoughts and feelings one may experience when having pain. Each item is scored on a 5-point scale (Sullivan et al. 1995).

#### Pain-free controls

Healthy subjects who participated in previous studies at the UF PAIN laboratory and had previously consented for the reuse of their data for future studies were eligible to be included as controls. Control participants were defined as: 1) participants without reporting chronic pain on most days within the past 6 months using the Graded Chronic Pain Scale, and 2) had also MRIs obtained using the same scanner and MRI protocol as the TN participants. To match a TN participant to a control, we selected a random participant in our TN sample and matched them with the closest same sex participant in age from our control dataset. This procedure was then repeated for the rest of the participants excluding the ones already selected (i.e., without replacement). To ensure enough matching controls across all age ranges, the procedure prioritized those age groups where our control dataset had fewer participants. Age ranges with a larger participant pool were addressed last. This approach was adopted to prevent a potential shortage of matching controls in the less-represented age groups. Previous studies were approved by UF IRB01.

### MRI data for TN and controls

All MRI data was collected at the UF MBI using a 3-Tesla Achieva Phillips (Best, the Netherlands) scanner using a 32-channel radio-frequency coil. A high resolution, T1-weighted (T1w) turbo field echo anatomical image was collected with TR = 7.0 ms, TE = 3.2 ms, 176 slices acquired in a sagittal orientation, flip angle = 8 degrees, resolution = 1 mm^3^. The TFE sequence used magnetization-prepared inversion recovery with an inversion time (TI) of 875 ms and inversion flip angle of 180°. Head movement was minimized via cushions positioned inside the head coil. Vital signs (blood pressure, temperature, and pulse) were also recorded prior to scanning.

### Brain age estimations

Brain age was estimated using two methods that have proven to be sensitive to chronic pain presence and severity (Cruz-Almeida et al., 2019; Johnson et al., 2022; Hung et al., 2022; Valdes-Hernandez et al., 2023; Valdes-Hernandez et al., 2024). The first one was BrainageR, a machine learning pipeline that applies Gaussian Process Regression (GPR) to structural MRI data (Cole et al., 2017). BrainageR was pretrained on a large sample of T1w MRIs of healthy individuals. The training dataset included more than 3000 healthy participants aged 18–92 years, aggregated from publicly available datasets such as IXI, OASIS, and ABIDE. T1w images were first processed using SPM12′s Unified Segmentation to classify tissues into gray matter, white matter, and cerebrospinal fluid (Ashburner & Friston, 2005). These segmentations were then nonlinearly normalized to MNI space using DARTEL (Ashburner, 2007). The resulting normalized tissue maps were vectorized and used as input features for the pretrained GPR model to generate brain age predictions. The BrainageR pipeline is available at https://github.com/james-cole/brainageR.”.

The second brain age prediction method employed was DeepBrainNet, a convolutional neural network recently developed to predict brain age (Bashyam et al., 2020). It was trained using slices of the T1w MRI images from 11,729 individuals (ages 3–95 years) from a diverse range of geographic locations, scanners, acquisition protocols, and studies, and tested in an independent sample of 2,739 individuals. Features for the DeepBrainNet are calculated as follows. First, the T1w needs to be skull-stripped. Second, the skull-stripped image has to be spatially normalized to the 1-mm isotropic voxel FSL skull-stripped T1w template using a 12-parameter linear affine transformation. For training, each of skull-stripped MRI image was divided into 80 2D slices (centered on the z = 0 plane in MNI coordinates) and considered as an independent sample. To obtain a final age prediction for a test sample, each of 80 slices of the test scan is input to the trained model independently and the median prediction is calculated as the subject’s predicted brain age. To obtain skull-stripped images in our sample, we used smriprep. Briefly, the T1w image was corrected for intensity non-uniformity using N4BiasFieldCorrection (Tustison et al., 2010) distributed with ANTs 2.2.0 and skull-stripped with a Nipype implementation of the antsBrainExtraction.sh workflow from ANTs (Klein et al., 2008), using OASIS30ANTs as target template.

The predicted brain age difference (brain-PAD) sometimes also called brain age gap (BAG) is the difference between a predicted brain age from an MRI and the person’s chronological age, and much research suggests its potential clinical value. Its utility to compare the degree of brain age acceleration among groups is straightforward. However, interpretability of absolute values, i.e., mean brain-PAD within a group, is obscured by the so-called “regression dilution” bias, which overestimates younger ages and underestimates older ages. This precludes us to understand which groups are actually experiencing accelerated brain aging and thus driving the group differences in brain-PAD. To report the clinically relevant unbiased brain-PADs, we recently advocated (Valdes-Hernandez, Laffitte Nodarse, Peraza, et al. 2023a; Valdes-Hernandez, Laffitte Nodarse, Cole, et al. 2023b) for the use of the correction of the predicted brain ages proposed by (Cole et al. 2018)

$$unbiased\;brain\;age=\frac{brain\;age-{\hat{\beta }}_{intercept}}{{\hat{\beta }}_{linear}}$$

In this equation, ${\hat{\beta }}_{intercept}$ and ${\hat{\beta }}_{linear}$ are the coefficients of the linear fit brainagechronologicalage.

### Data and statistical analysis

The present study was powered based on our previous research (Cruz-Almeida et al. 2019; Valdes-Hernandez, Laffitte Nodarse, Johnson, et al. 2023) and others (Hung et al. 2022) where a sample size of 54 will provide over 90 % power to detect a difference between two groups with two covariates at an alpha of 0.05. Missing data was treated using pairwise elimination.

For linear fits (i.e., ANCOVAs), to ensure normality, for every model (which entailed a specific subset of the whole dataset), we applied a rank-based inverse normal transformation to the dependent variable (brain-PAD) using the ‘Blom’ method with parameter c = 3/8 (Downton and Blom 1961). After fitting linear models, we applied the Shapiro-Wilk test of composite normality (with unspecified mean and variance) on the residuals (for Platykurtic distributions; while the Shapiro-Francia test was used for Leptokurtic distributions) to test whether the normality assumption required for linear models was fulfilled (Shapiro and Wilk 1965). All linear fits were re-run after removing those measurements deemed outliers. We deemed outliers as having their Cook’s distance, determined from the first regression, being 3 times higher than their sample average (Neter, 1990).

In the Bayesian analyses, Bayes factors were interpreted according to Jeffreys’ criterion (Jeffreys, 1961: no evidence (<1), anecdotal (1–3), moderate (3–10), strong (10–30), very strong (30–100), and decisive (>100) evidence for the alternative hypothesis. We also applied Kass and Raftery’s guideline (Raftery, 1995): no evidence (1), weak (1–3), positive (3–20), strong (20–150), and very strong (>150) evidence. For Bayes factors below 1, the reciprocal was used to categorize evidence in favor of the null hypothesis or exclusion.

#### Differences between groups in brain-PAD

To test whether there was a significant difference between groups in brain-PAD, we used Analysis of Covariance (ANCOVA) controlling for chronological age and sex. Thus, we tested the significance of the effect of the categorical variable group in the linear model brain-PAD ∼ group + sex + age, where sex took the values MALE and FEMALE, and age is the chronological age. To be consistent with the study by Hung and colleagues (Hung et al. 2022), we first performed the analysis with the nominal categorical variable group with levels CONTROLS and TN. We performed a second analysis including the categorical variable group with levels CONTROLS, Classical TN and Secondary/Idiopathic TN. Effects sizes were reported using the Cohen’s f^2^, which measures the relative variance explained by the effect when added to the regression model (0.1^2^ ≤ f^2^ < 0.25^2^ for small effects, 0.25^2^ ≤ f^2^ < 0.4^2^ for medium effects and f^2^ > 0.4^2^ for large effects. For pairwise comparisons, we additionally reported the difference in marginal means, namely ΔPAD, and its Standard Error (SE). Statistical significance was set to α = 0.05, with p-values corrected for multiple comparisons using a Bonferroni correction.

We applied a Bayesian framework to evaluate whether group effects contribute meaningfully to explaining variance in brain-PAD and to quantify the strength of that evidence. Bayesian linear regression was performed using JASP (version 0.19.3.0, JASP Team, Amsterdam, Netherlands), with dummy-coded TN group variables (namely ClassicalTN and SecIdioTN) as predictors, with controls as the reference level. Age and sex were included as nuisance covariates in all models, including the null model (H_0_: brain-PAD ∼ sex + age).

Model comparisons were conducted using Bayes factors (BF), which quantify the relative evidence for each model. The default Jeffreys–Zellner–Siow (JZS) prior was applied to the regression coefficients, with an r-scale parameter of 0.354, the default in JASP and recommended for small, expected effect sizes (Rouder et al., 2009). Bayes factors (BF_10_) were used to compare each model to the null model, while inclusion Bayes factors (BF_inclusion_) quantified how much the data supported the inclusion of each predictor. Bayesian Adaptive Sampling (BAS) was used as the default estimation method; results were validated using Markov Chain Monte Carlo (MCMC) with 10,000 posterior samples to confirm convergence and stability. The 95 % credible intervals were also reported for regression coefficients of the best-fitting model.

The specific R command was:

*jaspRegression::RegressionLinearBayesian(*.

*version = “0.19.2″,*

*formula = brain-* *PAD ∼ ClassicalTN.scale + SecIdioTN.scale + sex.scale + age,*

*isNuisance = ∼ sex.scale + age,*

*bayesFactorOrder = “nullModelTop”,*

*descriptives = TRUE,*

*effectsType = “matchedModels”,*

*modelsShown = “unlimited”,*

*posteriorSummaryTable = TRUE,*

 *summaryType = “best”)*.

#### Partial correlations between brain-PAD and clinical and psychological variables

Finally, to examine whether brain-PAD was associated with clinical and psychological symptoms, we employed partial correlations corrected for multiple comparisons (*q* < 0.05, one-sided, given our hypothesis was these variables were positively correlated with brain-PAD). Given there were significant differences between TN groups in BDI-II scores, BDI-II was included as a covariate in this analysis.

To assess whether these partial correlations were meaningful, a Bayesian correlation analysis was conducted also using JASP. A one-sided Bayesian Pearson correlation was computed between brain-PAD and the clinical or psychological variable, namely X—both previously adjusted for the covariates—testing the directional hypothesis that higher X was associated with higher brain-PAD values (i.e., a positive correlation). The Bayes factor for the directional hypothesis (BF_+0_) and the 95 % credible interval for the correlation coefficient was reported. The specific R command was:

jaspRegression::CorrelationBayesian(

 version = “0.19.2″,

 alternative = “greater”,

 ci = TRUE,

 pairwiseDisplay = TRUE,

 variablePairs = list(“adjusted brain-PAD”, “adjusted X”),

 variables = ∼ adjusted brain-PAD + adjusted X)

## Results

### Sample characteristics

The total sample is characterized in Table 1. In total, 55 TN participants gave written informed consent (69 % female, mean age ± standard deviation (SD) = 53.9 ± 14.9). One participant did not meet diagnosis criteria (n = 1) for either classical TN or secondary/idiopathic TN and was excluded from the study. The data from the remaining 54 TN participants, and the 54 sex- and age-matched controls were analyzed and are reported here. Specifically, 34 individuals were diagnosed with classical TN and 17 were diagnosed with secondary/idiopathic TN. In the Classical TN group, disease duration ranged from 1 to 31 years, while in secondary/idiopathic group, disease duration ranged from 1 to 15 years. However, the group difference in average disease duration was not statistically significant between those two groups (p = 0.066, see Table 1).

**Table 1:** Characteristics of the sample (n = 108).

		**Controls**	**Classical TN**	**Secondary/Idiopathic TN**	**Statistics**
Sex:	Females Males	38 16	23 14	15 2	χ^2^ = 3.80 p = 0.150
Age (years)		54.2 (17.4)	57.7 (13.6)	47.64 (14.2)	F_2,107_ = 2.37 p = 0.096
Disease Duration (years)		−	10.97 (7.54)	7.06 (5.07)	F_1,48_ = 3.53 p = 0.066
Usual Pain Intensity (0–100)		−	53.91 (36.53)	62.24 (32.86)	F_1,48_ = 0.62 p = 0.434
Pain Catastrophizing Scale		−	26.59 (12.59)	24.71 (11.42)	F_1,49_ = 0.27 p = 0.606
Pain Anxiety Symptom Scale		−	92.09 (33.46)	83.41 (30.42)	F_1,49_ = 0.81 p = 0.373
Beck Depression Inventory-II		−	11.33 (8.29)	19.12 (11.99)	F_1,47_ = 7.06 p = 0.011*

**Note:** Differences in categorical variables among groups were tested using the χ^2^; whereas differences in continuous variables were tested using ANOVA (Welch’s ANOVA yielded very similar results).

**Fig. 1:**
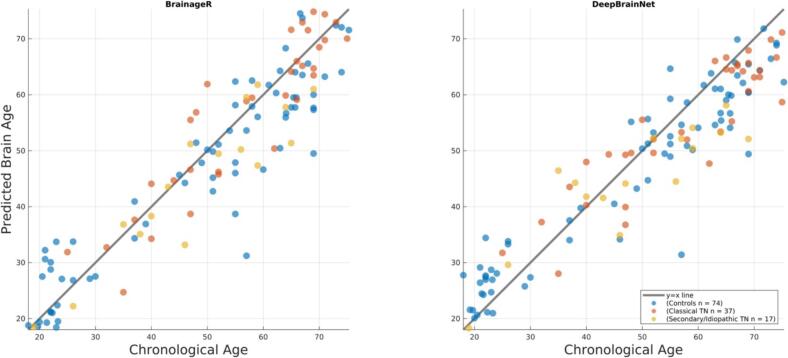
Brain age predictions versus chronological age for each group and brain age estimation method.

As expected, chronological age was highly correlated with the predicted brain age across all participants (p = 0.92, p ∼ 0, see Fig. 1 and Table 2**)**.

**Table 2:** Accuracy of brain age predictions for each group and the whole sample.

	Mean Absolute Error (years)		$r_{\mathrm{brainage},age}$		$R_{y=x}^{2}$	
	BaR	DBN	BaR	DBN	BaR	DBN
Controls	5.34 [4.39, 6.61]	5.32 [4.49, 6.52]	0.93 [0.89, 0.96]	0.94 [0.89, 0.97]	0.83 [0.71, 0.90]	0.81 [0.67, 0.88]
Classical TN	4.92 [3.78, 6.54]	5.34 [4.22, 6.75]	0.90 [0.81, 0.94]	0.90 [0.83, 0.94]	0.82 [0.66, 0.89]	0.67 [0.38, 0.82]
Secondary/ Idiopathic TN	5.09 [3.37, 7.48]	6.49 [4.41, 9.07]	0.92 [0.79, 0.97]	0.87 [0.66, 0.96]	0.74 [0.31, 0.93]	0.35 [-0.98, 0.86]
Whole sample	5.18 [4.49, 6.06]	5.48 [4.79, 6.29]	0.93 [0.89, 0.95]	0.93 [0.90, 0.95]	0.84 [0.77, 0.89]	0.77 [0.67, 0.84]

**Note:**$R_{y=x}^{2}=1-\frac{\sum {\left(y_{i}-x_{i}\right)}^{2}}{\sum {\left(y_{i}-\overset{¯}{y}\right)}^{2}}$, where $y_{i}$ and $x_{i}$ is the brain age and chronological age, respectively, for the i-th participant in the test data, i.e., assuming the “perfect” unbiased model y=x. The 95 % confidence interval (CI) of these accuracy measures was calculated using 10,000 bootstraps. BaR: BrainageR. DBN: DeepBrainNet.

### Differences in brain age acceleration between groups

#### ANCOVA effects

To be consistent with the study by Hung and colleagues (Hung et al., 2022), we first tested differences in brain-PAD between the TN and control groups. Brain-PAD, estimated with BrainageR, was 2.5 years greater in TN participants compared to controls (p = 0.037, effect size f^2^ = 0.2^2^), whereas no significant difference was detected when using DeepBrainNet.

**Fig. 2:**
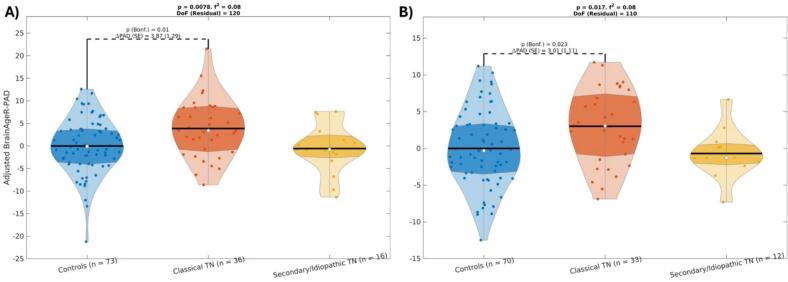
Differences in brain-PAD—estimated using BrainageR—between groups, adjusting for age and sex. A) Whole sample. B) After excluding outliers. Within the violin plots, the shaded area is the interquartile region, the white dot indicates the median and the black horizontal line is the mean. Effect sizes, as quantified by Cohen’s f2, are also shown. DoF: Degrees of Freedom. ΔPAD (in years): difference in brain-PAD across groups. SE (in years): Standard Error.

**Fig. 3:**
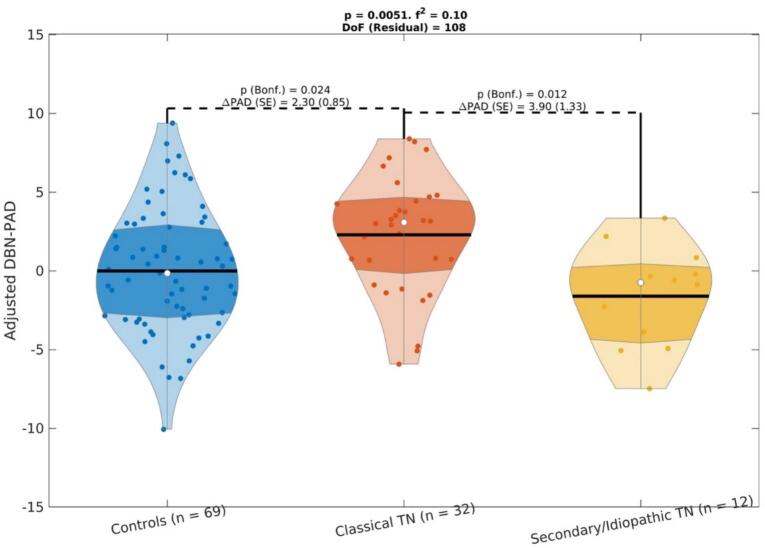
Differences in brain-PAD—estimated using DeepBrainNet—between groups, adjusting for age and sex, after excluding outliers (no significant results were found for the whole sample). Within the violin plots, the shaded area is the interquartile region, the white dot indicates the median and the black horizontal line is the mean. Effect sizes, as quantified by Cohen’s f2, are also shown. DoF: Degrees of Freedom. ΔPAD (in years): difference in brain-PAD across groups. SE (in years): Standard Error.

Specifically, there were significant differences in brain-PAD among TN subtypes where individuals with classical TN had significantly greater brain-PAD compared to controls by 3.9 years when using BrainageR in the whole sample (p = 0.01, Bonferroni-corrected across pairwise comparisons, Fig. 2A), by 3 years when using BrainageR after excluding outliers (p = 0.023, Bonferroni-corrected across pairwise comparisons, Fig. 2B), and by 2.3 years when using DeepBrainNet after excluding outliers (p = 0.024, Bonferroni-corrected across pairwise comparisons, Fig. 3).

We found no evidence of a significant difference in brain-PAD between secondary/idiopathic TN and controls, irrespective of the brain age estimation method or whether outliers were rejected or not. Moreover, the group by sex interaction was not statistically significant for any of these combinations either. For all the fits, rejection of composite normality of the residuals failed in all fits (p > 0.05).

#### Bayesian evidence for the group effects

Table 3 presents the BF_10_ values for each model, brain age estimation method, and sample (i.e., full sample vs. outlier-excluded), where the group effect was significant in the ANCOVA analysis. The model showing the strongest evidence, across all models and samples, relative to the null model (H_0_: brain-PAD ∼ covariates) was the one comparing the Classical TN group to the rest of the participants (H_1_: brain-PAD ∼ ClassicalTN + covariates), indicating strong or positive evidence in favor of this model. For this model, the BF_inclusion_ values indicated from moderate to strong (or positive) support for including ClassicalTN as a predictor of brain-PAD. On the other hand, the evidence for the model with the combined regressors (H_1_: brain-PAD ∼ ClassicalTN + covariates) was moderate for all models and samples, whereas there was anecdotal or weak evidence for the null when comparing the Idiopathic/Secondary TN group with the rest of the participants.

**Table 3:** Bayes Factors (B10) for the Bayesian Linear Regression.

**Model for H_1_**	Evidence Summary	BaR Whole sample	BaR excl. outliers	DBN Excl. outliers
brain-PAD ∼ ClassicalTN + SecIdioTN + covariates	BF_10_	6.5	3.0	4.3
		Moderate or positive for H_1_	Moderate or weak for H_1_	Moderate or positive for H_1_
brain-PAD ∼ SecIdioTN + covariates	BF_10_	0.4	0.4	0.8
		Anecdotal or weak for H_0_	Anecdotal or weak for H_0_	Anecdotal or weak for H_0_
brain-PAD ∼ ClassicalTN + covariates	BF_10_	22.1	10.0	11.3
		Strong for H_1_	Strong or positive for H_1_	Strong or positive for H_1_
	BF_inclusion_	14.4	6.6	7.0
		Strong and positive	Moderate or positive	Moderate or positive
	95 % Credible Interval (years)	[1.3, 6.0]	[0.8, 4.9]	[0.8, 4.1]

**Note:** H_0_: brain-PAD ∼ covariates. BaR: BrainageR. DBN: DeepBrainNet.

#### Unbiased brain-PAD values within groups

For the BrainageR method, the average (SE) of the unbiased brain-PAD (unbiased brain age minus chronological age) was −0.93 (0.82) years, 2.98 (1.21) years and −2.48 (1.46) years for the controls, classical TN, and secondary/idiopathic TN groups, respectively. For the DeepBrainNet method, these values were −0.59 (0.98) years, 5.61 (1.53) years and −2.34 (1.72) years, respectively. Using a one-sample *t*-test, we found evidence that the average unbiased brain-PAD was significantly different from zero for the classical TN group when using both the BrainageR (t_35_ = 2.47 with p = 0.019) and the DeepBrainNet (t_35_ = 3.67 with p = 0.0008) methods, whereas we did not find evidence that this measure was significantly different from zero in the other two groups. This suggests that the difference in brain-PAD is mainly driven by an older brain age in the classical TN group as seen in Fig. 2, Fig. 3.

### Association between brain age with clinical and psychological variables

**Fig. 4:**
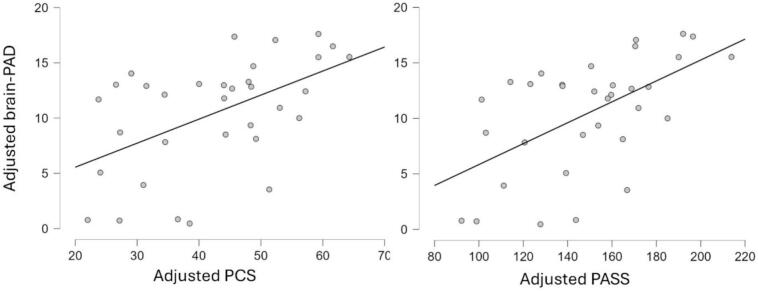
Scatter plots of brain-PAD estimated using DeepBrainNet, adjusted for age and sex versus the variables that were positively correlated it.

We ran partial correlations controlling for chronological age and sex within each TN group (significant associations are shown in Fig. 4). In individuals with classical TN, Brain-PAD estimated with DeepBrainNet—but not with BrainageR—had a significant positive association with PCS (r = 0.52, 95 % credible interval of [0.20, 0.72], p_+_ = 0.0014, q = 0.0028, with very strong evidence—or just strong according to Kass and Raftery’s guideline—BF_+0_ = 42.4) and PASS (r = 0.57, 95 % credible interval of [0.26, 0.75], p_+_ = 0.0004, q = 0.0018, with decisive evidence—or very strong according to Kass and Raftery’s guideline—BF_+0_ = 125.6); whereas no significant association between these variables were found in the secondary/idiopathic TN group. Additionally, no significant association was found between Brain-PAD—regardless of the brain age method—and disease duration or usual pain intensity in any TN group.

Furthermore, we compared these associations between TN groups for the variables that showed a significant relationship with Brain-PAD. Specifically, we tested whether the partial correlations between Brain-PAD (estimated with DeepBrainNet) and PCS or PASS differed significantly across groups by comparing their Fisher’s r-to-z–transformed coefficients using a Z-test, with the effective sample size adjusted (N minus the number of covariates) to account for the control variables. No significant differences were observed between groups. In addition, we tested the same group differences using an interaction model, brain-PAD ∼ sex + age + PCS (or PASS)*Group, and obtained similarly non-significant results for the PCS (or PASS):Group (interaction) term.

## Discussion

To our knowledge, this is the first study to examine a brain aging biomarker in persons with trigeminal neuralgia with consideration for the distinct types of trigeminal neuralgia diagnoses. Several important contributions emerged from this investigation. First, there were significant differences in brain-predicted age differences (brain-PAD or brain age gap) between individuals diagnosed with TN compared to age- and sex-matched pain-free controls. Second, those diagnosed with classical TN had “older” appearing brains compared to individuals with secondary/idiopathic TN or pain-free controls, regardless of age and sex. Finally, “older” brains were significantly associated with greater pain catastrophizing and pain-related anxiety levels in persons with TN accounting for age, sex, and depressive symptoms.

Consistent with our hypothesis, individuals diagnosed with TN had 2.5 years “older” brain relative to their own individual’s chronological age adjusting for important covariates. This is consistent with the only study to date that has examined brain aging processes in persons with TN (Hung et al., 2022). However, that study did not differentiate between TN diagnoses. In our current study, it appears that brain aging was mainly driven by individuals diagnosed with classical TN with almost 4 years “older” brains relative to their own individual’s chronological age on average. Previous data suggests that each extra year of brain-predicted age (i.e., having a brain-PAD score of +1) results in a 6.1 % relative subsequent increased mortality risk (Cole et al. 2018). Our findings also suggest that individuals diagnosed with secondary or idiopathic TN do not show accelerated brain aging processes as a group measured by our brain-wide brain aging biomarker. It is possible that in the classical TN presentation, the damage to the trigeminal nerve leads to brain aging processes either neuroinflammatory, neurodegenerative or both that are not a hallmark of secondary or idiopathic TN. This is currently speculative, and future studies both in rodents and humans are needed to elucidate the underlying brain aging mechanisms across the different TN subtypes as well as to test whether brain aging processes maybe antecedent or the result of the nerve compression.

An older brain age was also associated with greater pain catastrophizing and pain-related anxiety levels. This is consistent with our own previous work in older adults with musculoskeletal pain (Cruz-Almeida et al. 2019; Johnson et al., 2022). Interestingly, this remained statistically significant after accounting for differences in depressive symptoms, which is often co-morbid with pain, and anxiety across many chronic pain conditions. Taken together, our findings further support the importance of psychological characteristics in relation to brain structure and function that may be negatively impacted by chronic pain and be sensitive to our brain aging biomarker. Given that anxiety and depression have also been associated with accelerated brain aging (Johnson et al., 2022; Valdes-Hernandez et al., 2024) future research should attempt to disentangle these complex associations in persons with different TN diagnoses.

### Limitations and future directions

Our study has some limitations. While the sample size for the training set was large, the TN study cohort was relatively small. However, TN participants are well-characterized in their TN characteristics including a certified clinician examination and diagnosis. Second, the current analysis was cross-sectional; therefore, we cannot determine whether a specific brain-predicted age preceded or was subsequent to TN-associated pain. From the present findings, directionality or causality cannot be inferred as it is equally possible that brain aging plays a central role for the sensitivity and resilience to many symptoms and disorders associated with biological aging processes including TN. While accelerated brain aging may be a consequence of chronic pain, it is also plausible that specific brain aging patterns could represent a vulnerability factor predicting susceptibility to developing chronic pain conditions. Future longitudinal studies are needed to determine trajectories of brain aging and how they relate to TN and future health outcomes. Further, our brain aging biomarker does not provide the anatomical specificity to determine which brain regions are specifically “aged” as brain aging processes are not a uniform process across all individuals. This limitation is particularly relevant given that brain aging patterns likely vary across individuals with TN and its subtypes. The development of region-specific aging biomarkers represents an important future direction that could provide more personalized insights by pinpointing where accelerated aging is occurring in each individual, potentially helping the field better understand heterogeneity in pain susceptibility and treatment response. Finally, the unbalanced group sizes—particularly the underrepresentation of secondary/idiopathic TN patients in our sample—may limit the generalizability of our findings for this subgroup and reduce statistical power for detecting differences. Future studies including a larger number of individuals with secondary/idiopathic TN are needed to further elucidate these associations.

Our findings establish important foundational evidence for differential brain aging patterns between TN subtypes, which opens several critical avenues for future investigation. First, mechanistic studies incorporating multi-modal approaches—including blood-based biomarkers (e.g., cortisol, inflammatory cytokines, neurotrophic factors), cerebrospinal fluid analysis, and advanced neuroimaging techniques such as diffusion tensor imaging and functional connectivity—are needed to determine the cellular and molecular mechanisms driving these brain age differences. Second, emerging techniques such as single-cell RNA sequencing from post-mortem tissue, advanced MRI methods capable of cell-type specific imaging, and PET imaging with targeted tracers will be essential to identify the specific cell types (e.g., neurons, microglia, astrocytes, oligodendrocytes) affected by these neurobiological processes and to characterize the temporal patterns of these changes across disease progression. Third, comprehensive neuropsychological assessments, multi-modal MRI during pain and cognitive tasks, and longitudinal monitoring are required to determine whether these structural brain aging differences translate into measurable impacts on cognitive function, pain processing, emotional regulation, and quality of life outcomes. Finally, future studies might benefit from examining whether associations differ based on the magnitude or direction of brain-age differences. Such multi-faceted investigations will be crucial for developing targeted therapeutic interventions that address the underlying neurobiological mechanisms rather than solely focusing on symptom management, while ultimately advancing precision medicine approaches for individuals diagnosed with TN.

### Conclusions

We report here accelerated brain aging processes in individuals with classical TN, but not in persons diagnosed with secondary or idiopathic TN. Our study replicates previous findings in this population but adds to the literature that accelerated brain aging may not occur across all subtypes. Given the increased use of MRI for TN diagnostics, combined with our own recent work deriving brain aging biomarker from clinical-grade scans (Valdes-Hernandez et al., 2023a, Valdes-Hernandez et al., 2023b), future studies within clinical settings are feasible and needed to truly understand this debilitating complex condition.

## CRediT authorship contribution statement

**Yenisel Cruz-Almeida:** Writing – review & editing, Writing – original draft, Supervision, Project administration, Methodology, Investigation, Funding acquisition, Data curation, Conceptualization. **Pedro A. Valdes-Hernandez:** Writing – review & editing, Software, Methodology, Formal analysis, Data curation. **Yun Liang:** Writing – review & editing, Software, Data curation. **Mingzhou Ding:** Writing – review & editing, Supervision, Software, Project administration, Methodology, Investigation, Data curation. **John K. Neubert:** Writing – review & editing, Supervision, Project administration, Investigation.

## Declaration of competing interest

The authors declare that they have no known competing financial interests or personal relationships that could have appeared to influence the work reported in this paper.

## Data Availability

Data will be made available on request.
